# Prospective Observational Study of Safety of Early Treatment with Edoxaban in Patients with Ischemic Stroke and Atrial Fibrillation (SATES Study)

**DOI:** 10.3390/brainsci11010030

**Published:** 2020-12-30

**Authors:** Giovanni Frisullo, Paolo Profice, Valerio Brunetti, Irene Scala, Simone Bellavia, Aldobrando Broccolini, Pietro Caliandro, Riccardo Di Iorio, Roberta Morosetti, Fabio Pilato, Renzo Laborante, Giacomo Della Marca

**Affiliations:** 1 Dipartimento Scienze dell’Invecchiamento, Neurologiche, Ortopediche e della Testa-Collo, Fondazione Policlinico Universitario A. Gemelli IRCCS, Largo Agostino Gemelli, 8, 00168 Rome, Italy; v.brunetti2509@gmail.com (V.B.); Aldobrando.broccolini@policlinicogemelli.it (A.B.); pietro.caliandro@policlinicogemelli.it (P.C.); riccardo.diiorio@policlinicogemelli.it (R.D.I.); roberta.morosetti@policlinicogemelli.it (R.M.); fabio.pilato@policlinicogemelli.it (F.P.); Renzo.laborante@libero.it (R.L.); giacomo.dellamarca@policlinicogemelli.it (G.D.M.); 2 Neurology Unit Mater Olbia Hospital, 07026 Olbia Sassari, Italy; paolo.profice@policlinicogemelli.it; 3 School of Medicine and Surgery, Catholic University of Sacred Heart, Largo Francesco Vito, 1, 00168 Rome, Italy; irene.scala92@gmail.com (I.S.); bellavia.sim@gmail.com (S.B.)

**Keywords:** stroke, anticoagulant, safety, bleeding, non-valvular atrial fibrillation

## Abstract

New direct oral anticoagulants are recommended for stroke prevention in patients with non-valvular atrial fibrillation (NVAF). However, no data are available regarding the optimal time to start oral anticoagulation after acute stroke. The aim of our study was to evaluate the occurrence of symptomatic bleedings within 90 days from acute cardioembolic stroke in patients who received early treatment with Edoxaban. The study was conducted according to an observational prospective uncontrolled design. Secondary endpoints were the incidence of major bleeding (MB), hemorrhagic transformation within the first week of Edoxaban treatment, minor bleeding, and recurrent stroke. We included patients with Alberta Stroke Program Early Computed Tomography Score (ASPECTS) ≥ 6, NVAF, no previous treatment with any other anticoagulant, preserved swallowing function. Patients with estimated Glomerular Filtration Rate < 50 mL/min, body weight < 60 kg, receiving cyclosporine, dronedarone, erythromycin, ketoconazole, or previous treatment with any other anticoagulant were excluded. We enrolled 75 elderly patients with moderate disability. We did not observe any symptomatic intracranial bleeding or recurrent stroke after 3 months of treatment with early administration of Edoxaban, while two gastrointestinal MB, and 11 minor bleedings were reported. Asymptomatic bleeding was evaluated with a brain Magnetic Resonance Imaging performed 5 days after starting anticoagulant treatment with Edoxaban. Specifically, we observed small petechiae in 12% of the patients, confluent petechiae in 6.6% of the patients, and small hematoma of the infarcted area in 2.7% of the patients. No intralesional hematoma or hemorrhagic lesion outside the infarcted area were observed. According to our data, the early use of Edoxaban seems to be safe in patients after cardioembolic stroke. However, due to the small size of the study sample, and the short follow-up period, further studies are needed.

## 1. Introduction

Non-valvular atrial fibrillation (NVAF) is the most common cause of cardioembolic stroke, which is the most severe ischemic stroke subtype [1]. Cardioembolic stroke is characterized by increasing prevalence, early and high rate of recurrence and severe disability and high mortality, thus, appropriate antithrombotic prophylaxis should be started as soon as possible [2]. However, there is no accordance about the time to start anticoagulant therapy for secondary prevention after acute ischemic stroke in patients with NVAF, due to the potential hemorrhagic transformation risk [3,4,5,6,7,8]. Thus, despite its enormous preventive potential, oral anticoagulation is not prescribed or often postponed, exposing patient to an increased risk of stroke recurrence. The reason for this treatment delay is often the concern of brain bleeding at the site of the ischemic lesion. To date, there is few and poor evidence about the optimal time for starting new oral anticoagulants (NOACs) in patients with acute ischemic stroke [3,4,5,6,7,8]. Edoxaban is an oral anti-factor Xa anticoagulant, approved for use in the prevention of stroke in patients with NVAF and in the treatment of acute venous thromboembolism [9]. Although the safety profile of Edoxaban seems to be superior to warfarin, there are no data regarding the safety of early administration of Edoxaban in patients with acute ischemic stroke [10]. The principal aim of the study was to evaluate the occurrence of symptomatic bleedings within 90 days from acute cardioembolic stroke in patients who received early treatment with Edoxaban. As secondary aims, we evaluated the occurrence of hemorrhagic infarction (HI) and parenchymal hematoma (PH) during the first week of Edoxaban treatment, by brain computed tomography (CT) or Magnetic Resonance Imaging (MRI).

## 2. Materials and Methods

### 2.1. Study Design and Population

In this prospective, non-randomized, uncontrolled, single center study, we included patients starting anticoagulant treatment with Edoxaban at therapeutic dose (60 mg/die) within five days from cardioembolic stroke onset. All patients underwent a CT scan 24 h after the acute stroke and continuous clinical and multimodal monitoring in Stroke Unit (Fondazione Policlinico Universitario Agostino Gemelli—Catholic University—Rome, Italy). During hospitalization, 5 ± 2 days after Edoxaban initiation, all patients underwent brain MRI to detect hemorrhagic transformation (HT) or other intracranial bleeding. Only patients who provided a written informed consent and signed the ethics committee-approved informed consent form were eligible for screening. To be enrolled into the study, patients had to be at least 18 years of age and affected by NVAF and acute stroke with Alberta Stroke Program Early CT Score (ASPECTS) or Posterior Circulation Acute Stroke Prognosis Early CT Score (pc-ASPECTS) ≥ 6 at baseline CT scan [11,12]. Exclusion criteria were: (1) acute or chronic renal failure, defined as estimated Glomerular Filtration Rate < 50 mL/min (Cockcroft Gault formula), (2) body weight < 60 kg, (3) ongoing treatment with cyclosporine, dronedarone, erythromycin, ketoconazole, (4) known hypersensitivity to Edoxaban, (5) or contraindication to treatment with an anticoagulant. The study protocol was approved by the Ethical committee of Fondazione Policlinico Universitario “A Gemelli” IRCCS—Rome (Prot. 49434/17 ID:1797).

### 2.2. Clinical Assessment

The following demographic and clinical variables were recorded during the acute phase: age, sex, hypertension, diabetes mellitus, dyslipidemia, smoking, prior stroke/ transient ischemic attack (TIA), previous vascular diseases, previous antiplatelet treatment, CHA2DS2-VASc (congestive heart failure, hypertension, age ≥ 75 years, diabetes mellitus, stroke or TIA, vascular disease, age 65 to 74 years, sex category) score, HAS-BLED (hypertension, abnormal renal/liver function, stroke, bleeding history or predisposition, labile international normalized ratio, age ≥ 75 years, drugs/alcohol concomitantly) score, type of NVAF, Oxfordshire Community Stroke Project (OCSP) classification, wake-up stroke, National Institute of Health Stroke Scale (NIHSS) score at admission, NIHSS score after revascularization, NIHSS score at discharge, Glasgow Coma Scale (GCS), thrombolysis, thrombectomy, ASPECTS/pc-ASPECTS (based on TC scan performed 24 h after acute stroke), HT prior and after Edoxaban initiation, HT subtype. At three months follow-up, the following data were collected: intracranial bleeding, major and minor bleeding, blood transfusion, hospitalization, deaths, modified Rankin Scale (mRS) score, adherence to treatment, stroke recurrence, and myocardial infarction. Major bleeding included fatal bleeding and/or symptomatic bleeding in a critical area or organ, such as intracranial, intraspinal, intraocular, retroperitoneal, intraarticular or pericardial, or intramuscular with compartment syndrome, and/or bleeding causing a fall of the hemoglobin level by at least 20 gr per liter, need of a transfusion of at least 2 units of blood, or symptomatic bleeding of an organ or critical area [13].

### 2.3. CT/MRI Assessment

HT was categorized into four different subtypes without taking into consideration whether or not it was associated with clinical deterioration. HT was categorized according to following definitions: hemorrhagic infarction type 1 (HI-1) was defined as small petechiae along the margins of the infarct, and HI type 2 (HI-2) was defined as more confluent petechiae within the infarcted area but without space-occupying effect; parenchymal hematoma type 1 (PH-1) was defined as hematoma in ≤30% of the infarcted area with some slight space-occupying effect, and parenchymal hematoma type 2 (PH-2) was defined as dense hematoma > 30% of the infarcted area with substantial space-occupying effect or as any hemorrhagic lesion outside the infracted area. Symptomatic hemorrhagic transformation was defined as PH2 associated with clinical deterioration with the increase of at least 4 points on the NIHSS [14,15].

### 2.4. Statistical Analysis

Given the exploratory nature of this study, no sample size analysis was performed. We considered a number of 75 patients as a potentially informative sample for an observational prospective safety study. For continuous measures, medians and interquartile ranges (IQR) are presented; for categorical measures, frequencies and percentages are presented. On univariate analysis, the Mann–Whitney U-test was used to compare continuous variables between patients with and without HT (HT+ group and HT− group, respectively) after starting Edoxaban therapy; the Pearson’s χ^2^ was used to compare categorical variables between the two groups. A value of *p* < 0.05 was considered significant. Statistical analyses were performed using Statistical Package for Social Science (SPSS) software version 20.

## 3. Results

**Figure 1:**
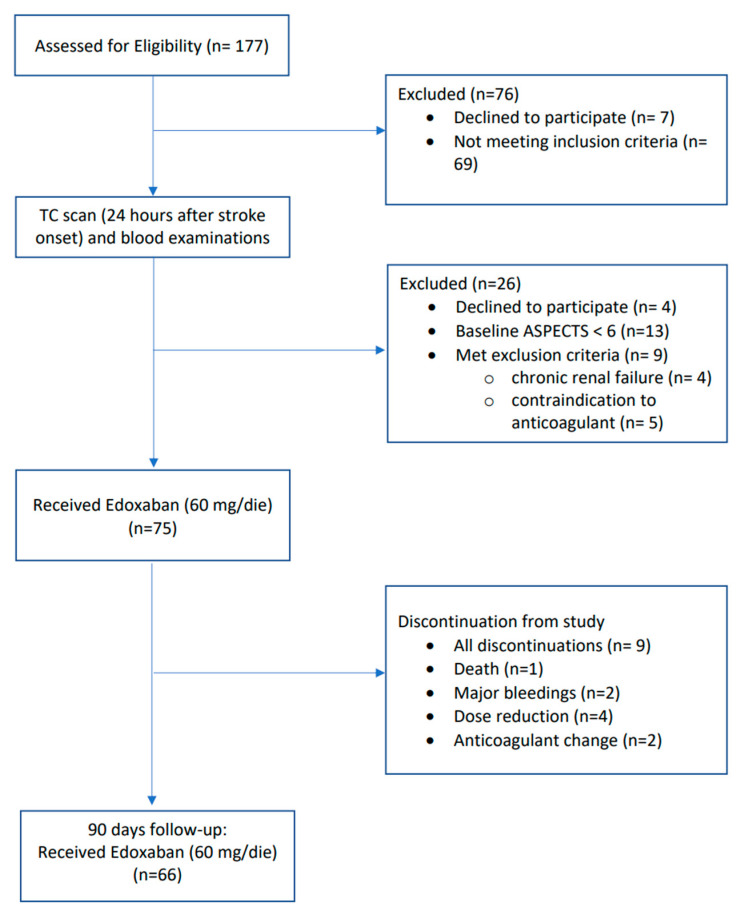
The CONsolidated Standards of Reporting Trials (CONSORT) flow diagram of the study showing data on enrolment, number of patients at different time points, withdrawal from the study, and mortality.

Between 1 November 2017 and 31 December 2018, 177 patients were assessed for study eligibility. Of those screened, 76 patients were excluded because they did not meet inclusion criteria (*n* = 69), or declined to participate to the study (*n* = 7). After CT scan and blood examinations performed 24 h after stroke unset, 26 patients were excluded because of ASPECTS < 6 (*n* = 13), withdrawal of consent (*n* = 4), or presence of exclusion criteria (*n* = 9). The data on enrollment, number of patients at different time points in clinical and imaging follow-ups, dropout from the study and mortality are summarized in the CONsolidated Standards of Reporting Trials (CONSORT) flow diagram of the study (Figure 1).

Seventy-five patients (median age: 78.3 years; 48 female, 27 male) were enrolled in the Prospective Observational Study of Safety of Early Treatment with Edoxaban in Patients with Ischemic Stroke and Atrial Fibrillation (SATES) study. Arterial hypertension was the prevalent medical condition (73%), followed by hyperlipidemia (23%), diabetes (20%), and smoking habit (17%). Mean CHA2DS2-VASc score was 5.2, indicating a moderate-high risk for cardioembolic stroke, and mean HAS-BLED was 3. Upon admission, atrial fibrillation was frequently unknown (61%). No recruited patient had ever taken anticoagulant therapy before this stroke while 26.7% of patients was on antiplatelet therapy. Demographic and baseline features are summarized in Table 1.

Based on the OCSP classification 56 (78%) patients showed neurological signs compatible with partial anterior circulation infarcts (PACI), 15 (20%) patients with posterior circulation infarcts (POCI), and four (5.3%) lacunar infarcts (LACI), while no patients presented total anterior circulation infarct (TACI). Median NIHSS score was six at the admission and two at discharge, consistent with an improvement of the neurological deficit during the hospitalization. Twenty-eight patients (37.3%) underwent thrombolysis and 17 (22.7%) underwent thrombectomy. The median time of therapy start with Edoxaban after the stroke onset was 2.0 days (IQR = 2.0). Detailed demographic and baseline features are summarized in Table 1.

Brain MRI performed 5 ± 2 days after starting treatment with Edoxaban showed asymptomatic hemorrhagic transformation in 16 (21%) patients: nine (12%) patients presented HI-1, 5 (6.6%) HI-2, and two (2.7%) PH-1. No patient presented a PH-2 (Table 2).

At 3 months, none of the patients had major intracranial bleeding, two (2.7%) patients presented major extracranial bleedings (gastrointestinal), and 11 (14.7%) patients presented minor bleeding (five epistaxis, three gingival bleedings, and three cutaneous hematoma). Excellent outcome at 3 months (defined as mRS 0-1) was observed in 53 (70.1%) patients. One death from sepsis was observed within 3 months in all the study population. In six patients, we observed a lack of adherence to the treatment due in four cases to a reduction of the dose for minor bleeding and in the other two cases to a change of the oral anticoagulant made by cardiologist. Detailed clinical data and outcomes are reported in Table 3.

Comparing patients with and without asymptomatic HT we did not find significant difference in demographic, clinical or neuroradiological features except for a significantly higher NIHSS at time of discharge (HT+ group: 4.4 ± 3.3 vs. HT− group: 2.3 ± 3.1; *p* = 0.011; Mann–Whitney U = 590.000), and a higher prevalence of wake-up stroke (HT+ group: 35.7% vs. HT− group: 13.3%; *p* = 0.048; χ^2^ = 3.926) in HT group.

## 4. Discussion

NOACs prescription during in-hospital stay after stroke is common in clinical practice [16]. However, the optimal timing for starting or resuming NOAC therapy after a cardioembolic stroke is still uncertain because of safety concern due to its potential hemorrhagic risk. Large prospective randomized trials, investigating early versus late initiation of NOAC administration in patients with atrial-fibrillation related stroke (Early Versus Late Initiation of Direct Oral Anticoagulants in Post-Ischaemic Stroke Patients with Atrial Fibrillation (ELAN): An International, Multicentre, randomised-Controlled, Two-Arm, Assessor-Blinded Trial, ELAN Trial [17], Optimal Timing of Anticoagulation after Acute Ischaemic Stroke, OPTIMAS Trial [18], Timing of Oral Anticoagulant Therapy in Acute Ischemic Stroke with Atrial Fibrillation, TIMING trial [19], Optimal Delay Time to Initiate Anticoagulation after Ischemic Stroke in Atrial Fibrillation, START Trial [20], Lixiana Acute Stroke Evaluation Registry, LASER [21], and Apixaban for Early Prevention of Recurrent Embolic Stroke and Hemorrhagic Transformation, AREST trial [22]) are ongoing. We designed a prospective, non-randomized, uncontrolled, single center study to evaluate the safety of very early initiation of Edoxaban in a selected population of acute ischemic stroke patients. No symptomatic intracranial bleeding occurred in the first 3 months of treatment. The only major bleedings we observed were gastrointestinal and portal hemorrhage leading only to a temporary interruption of Edoxaban. However, this extracranial event was not related to the early beginning of therapy with a NOAC because of its late occurrence (2 months after Edoxaban administration). On the other hand, major gastrointestinal bleeding has been described in elderly patients in some NOACs trials with a higher percentage than that observed in our study [23,24,25]. In particular, in the Global Study to Assess the Safety and Effectiveness of Edoxaban (DU-176b) vs Standard Practice of Dosing With Warfarin in Patients With Atrial Fibrillation (ENGAGE-TIMI 48) trial the rate of major gastrointestinal bleeding in the high-dose Edoxaban group was higher (3.3%) than in our cohort, probably due to the longest follow-up period [23]. In the three months follow-up, seven patients (16%) presented minor bleeding; no one needed discontinuation of Edoxaban and in only one case a reduction of dose was necessary. On the contrary, in the ENGAGE-TIMI 48 trial the rate of minor bleedings after 3–12 months from standard dose of Edoxaban introduction was 8.6% [23]. The higher incidence of minor bleedings in our cohort is probably due to the baseline selection of patients, since in the ENGAGE-TIMI 48 all patients with high bleeding risk or acute cardiovascular and cerebrovascular ischemic events were excluded. In the short-term radiological follow-up (5 ± 2 days from the beginning of anticoagulant therapy) we found HT in 16 patients (21%). All these intracranial bleedings were asymptomatic, and no one required an interruption of treatment with Edoxaban. The HT rate we found in our cohort was similar to that observed in in other clinical trials and in one observational study [26,27,28]. Therefore, treatment with Edoxaban does not seem to influence the hemorrhagic transformation of the acute ischemic lesion. Overall, the higher NIHSS at discharge of HT group suggest that the presence of a hemorrhagic infarction, even petechial, influences the short-term recovery capabilities, without affecting the long-term disability. Despite the high risk of cardioembolic brain events (mean CHADsVASC score = 5.2 ± 1.4), no patient presented a recurrence of ischemic stroke or a new systemic thromboembolic event in the 3 month follow-up. In three observational studies evaluating the rate of stroke recurrence after an early introduction of NOACs or VKA therapy (median time 5 days) [28], the rate of recurrent stroke in high cardioembolic risk patients (CHADsVASC from 5 to 6) during a 3 month follow-up period ranged from 2% to 2.7% [4,6,28]. The higher rate of stroke recurrence observed in these studies as compared to our cohort could be due to the sample size or to the specificity of the single treatment. Although the patients included in this study fell into a moderate risk bleeding category (median HASBLED score = 3), further studies will be needed to evaluate the safety of Edoxaban treatment in patients with more extensive ischemic injury (ASPECT < 6).

## 5. Conclusions

The small size of the study sample, the short follow-up period, and the absence of a control group with delayed anticoagulant treatment are the main limitations of our study, and further (and still pending) clinical trials will be useful to clarify any doubts. However, our data suggest that early administration of Edoxaban therapy, within five days from stroke onset, in a selected cohort of anticoagulant-naïve patients with NVAF associated ischemic stroke, and an ASPECT score ≥ 6, might be safe.

## Figures and Tables

**Table 1:** Demographic features, risk factors, stroke classification, clinical assessment, and procedures of patients included in the study.

Demographic Features and Risk Factors	*n* (%)
Age, median (y, IQR)	78.3 (13.5)
Gender, female, *n* (%)	48 (64)
**Risk factors**	
Hypertension, *n* (%)	55 (73.3)
Diabetes mellitus, *n* (%)	15 (20)
Hyperlipidemia, *n* (%)	17 (22.7)
Cigarette smoking, *n* (%)	13 (17.3)
Previous stroke/TIA, *n* (%)	14 (18.6)
Previous vascular disease, *n* (%)	14 (18.6)
Antiplatelet therapy prior to stroke, *n* (%)	20 (26.7)
CHA2DS2VASc score, median (IQR)	5 (2)
HASBLED score, median (IQR)	3 (0)
**Atrial fibrillation**	
Paroxysmal, *n* (%)	27 (36)
Persistent, *n* (%)	2 (2.7)
Permanent, *n* (%)	11 (14.7)
Unknown, *n* (%)	35 (46.7)
**OCSP ^1^ classification**	
PACI ^2^, *n* (%)	56 (74.7)
POCI ^3^, *n* (%)	15 (20)
LACI ^4^, *n* (%)	4 (5.3)
TACI ^5^, *n* (%)	0 (0)
**Clinical assessment and procedures**	
Wake-up stroke, *n* (%)	14 (18.9)
NIHSS ^6^ at stroke onset, median (IQR)	6 (8)
NIHSS after revascularization, median (IQR)	5 (5.5)
NIHSS after discharge, median (IQR)	2 (5)
GCS ^7^ at stroke onset, median (IQR)	15 (1)
Thrombolysis, *n* (%)	28 (37.3)
Thrombectomy, *n* (%)	17 (22.7)

^1^ OSCP: The Oxfordshire Community Stroke Project. ^2^ PACI: Partial Anterior Cerebral Infarction; ^3^ POCI: Posterior Cerebral Infarction; ^4^ LACI: Lacunar Cerebral Infarction; ^5^ TACI: Total Anterior Cerebral Infarction; ^6^ NIHSS: National Institute of Health Stroke Scale; ^7^ GCS: Glasgow Coma Scale.

**Table 2:** Neuroradiological Features of Patients Included in the Study.

Neuroradiological Features	
ASPECT ^1^ at Emergency Department CT scan, median (IQR)	10 (1)
HT ^2^ at Emergency Department CT scan, *n* (%)	2 (2.7)
HT subtypes, (*n*)	HI-1 ^3^ (1), HI-2 ^4^ (1), PH-1 ^5^ (0), PH-2 ^6^ (0)
ASPECT after 24 h from the stroke onset, median (IQR)	8 (1)
HT on brain MRI, 5 days after stroke onset, *n* (%)	16 (21.3)
HT subtypes, (*n*)	HI-1 (9), HI-2 (5), PH-1 (2), PH-2 (0)

^1^ Alberta stroke program early CT; ^2^ Hemorrhagic transformation; ^3^ Hemorrhagic infarction type 1—small petechiae; ^4^ Hemorrhagic infarction type 2—confluent petechiae; ^5^ Parenchymal hematoma type 1—hematoma in <30% of the infarcted area, with a mild space-occupying effect); ^6^ PH-2 (hematoma in >30% of the infarcted area, with a significant space-occupying effect.

**Table 3:** Adverse events, hospitalization, and death after 3 months from the inclusion in the study.

Clinical Evaluation at 3 Months Follow-Up	
Intracranial bleedings, *n* (%)	0 (0)
Major bleedings, *n* (%)	2 (2.7)
Minor bleedings, *n* (%)	11 (14.7)
Blood transfusion, *n* (%)	0 (0)
Hospitalization, *n* (%)	4 (5.4)
Deaths, *n* (%)	1 (1.3)
mRS ^1^, median (IQR)	1 (2)
Adherence to treatment, *n* (%)	69 (92)
Stroke recurrence, *n* (%)	0 (0)
Myocardial infarction, *n* (%)	0 (0)

^1^ Modified Rankin Scale.

## Data Availability

The data presented in this study are available on request from the corresponding author. The data are not publically available due to ethical restrictions.

## References

[1] Odutayo A., Wong C.X., Hsiao A.J., Hopewell S., Altman D.G., Emdin C.A. (2016). Atrial fibrillation and risks of cardiovascular disease, renal disease, and death: Systematic review and meta-analysis. BMJ.

[2] Paciaroni M., Agnelli G., Falocci N., Caso V., Becattini C., Marcheselli S., Rueckert C., Pezzini A., Poli L., Padovani A. (1995). Early Recurrence and Cerebral Bleeding in Patients with Acute Ischemic Stroke and Atrial Fibrillation: Effect of Anticoagulation and Its Timing: The RAF Study. Stroke.

[3] Klijn C.J., Paciaroni M., Berge E., Korompoki E., Kõrv J., Lal A., Putaala J., Werring D.J. (2019). Antithrombotic treatment for secondary prevention of stroke and other thromboembolic events in patients with stroke or transient ischemic attack and non-valvular atrial fibrillation: A European Stroke Organisation guideline. Eur. Stroke J..

[4] Arihiro S., Todo K., Koga M., Furui E., Kinoshita N., Kimura K., Yamagami H., Terasaki T., Yoshimura S., Shiokawa Y. (2016). Three-month risk-benefit profile of anticoagulation after stroke with atrial fibrillation: The SAMURAI-Nonvalvular Atrial Fibrillation (NVAF) study. Int. J. Stroke.

[5] Gioia L.C., Kate M., Sivakumar L., Hussain D., Kalashyan H., Buck B., Bussiere M., Jeerakathil T., Shuaib A., Emery D. (2016). Early Rivaroxaban Use After Cardioembolic Stroke May Not Result in Hemorrhagic Transformation: A Prospective Magnetic Resonance Imaging Study. Stroke.

[6] Seiffge D.J., Traenka C., Polymeris A., Hert L., Peters N., Lyrer P., Engelter S.T., Bonati L.H., De Marchis G.M. (2016). Early start of DOAC after ischemic stroke: Risk of intracranial hemorrhage and recurrent events. Neurology.

[7] Paciaroni M., Angelini F., Agnelli G., Tsivgoulis G., Furie K.L., Tadi P., Becattini C., Falocci N., Zedde M., Abdul-Rahim A.H. (2017). Prediction of early recurrent thromboembolic event and major bleeding in patients with acute stroke and atrial fibrillation by a risk stratification schema: The ALESSA score study. Stroke.

[8] Wilson D., Ambler G., Banerjee G., Shakeshaft C., Cohen H., Yousry T.A., Al-Shahi Salman R., Lip G., Houlden H., Brown M.M. (2019). Early versus late anticoagulation for ischaemic stroke associated with atrial fibrillation: Multicentre cohort study. J. Neurol. Neurosurg. Psychiatry.

[9] Bounameaux H., Camm A.J. (2014). Edoxaban: An update on the new oral direct factor Xa inhibitor. Drugs.

[10] Goette A., Merino J.L., Ezekowitz M.D., Zamoryakhin D., Melino M., Jin J., Mercuri M.F., Grosso M.A., Fernandez V., Al-Saady N. (2016). Edoxaban versus enoxaparin-warfarin in patients undergoing cardioversion of atrial fibrillation (ENSURE-AF): A randomised, open-label, phase 3b trial. Lancet.

[11] Barber P.A., Demchuk A.M., Zhang J., Buchan A.M. (2000). Validity and reliability of a quantitative computed tomography score in predicting outcome of hyperacute stroke before thrombolytic therapy. ASPECTS Study Group. Alberta Stroke Programme Early CT Score. Lancet.

[12] De Marchis G.M., Kohler A., Renz N., Arnold M., Mono M.L., Jung S., Fischer U., Karameshev A.I., Brekenfeld C., Gralla J. (2011). Posterior versus anterior circulation strokes: Comparison of clinical, radiological and outcome characteristics. J. Neurol. Neurosurg. Psychiatry.

[13] Schulman S., Kearon C. (2005). Definition of major bleeding in clinical investigations of antihemostatic medicinal products in non-surgical patients. J. Thromb. Haemost..

[14] Molina C.A., Alvarez-Sabín J., Montaner J., Abilleira S., Arenillas J.F., Coscojuela P., Romero F., Codina A. (2002). Thrombolysis-related hemorrhagic infarction: A marker of early reperfusion, reduced infarct size, and improved outcome in patients with proximal middle cerebral artery occlusion. Stroke.

[15] Trouillas P., von Kummer R. (2006). Classification and pathogenesis of cerebral hemorrhages after thrombolysis in ischemic stroke. Stroke.

[16] Haeusler K.G., Tütüncü S., Kunze C., Schurig J., Malsch C., Harder J., Wiedmann S., Dimitrijeski B., Ebinger M., Hagemann G. (2019). Oral anticoagulation in patients with atrial fibrillation and acute ischaemic stroke: Design and baseline data of the prospective multicentre Berlin Atrial Fibrillation Registry. Europace.

[17] Early Versus Late Initiation of Direct Oral Anticoagulants in Post-Ischaemic Stroke Patients with Atrial Fibrillation (ELAN): An International, Multicentre, randomised-Controlled, Two-Arm, Assessor-Blinded Trial (ELAN). https://clinicaltrials.gov/ct2/show/NCT03148457.

[18] Optimal Timing of Anticoagulation after Acute Ischaemic Stroke: A randomised Controlled Trial (OPTIMAS). https://clinicaltrials.gov/ct2/show/NCT03759938.

[19] Timing of Oral Anticoagulant Therapy in Acute Ischemic Stroke with Atrial Fibrillation. https://clinicaltrials.gov/ct2/show/NCT02961348.

[20] Optimal Delay Time to Initiate Anticoagulation after Ischemic Stroke in Atrial Fibrillation (START). https://clinicaltrials.gov/ct2/show/NCT03021928.

[21] Lixiana Acute Stroke Evaluation Registry (LASER). https://clinicaltrials.gov/ct2/show/NCT03494530.

[22] Apixaban for Early Prevention of Recurrent Embolic Stroke and Hemorrhagic Transformation (AREST). https://clinicaltrials.gov/ct2/show/NTC02283294.

[23] Giugliano R.P., Ruff C.T., Braunwald E., Murphy S.A., Wiviott S.D., Halperin J.L., Waldo A.L., Ezekowitz M.D., Weitz J.I., Špinar J. (2013). Edoxaban versus warfarin in patients with atrial fibrillation. N. Engl. J. Med..

[24] Connolly S.J., Ezekowitz M.D., Yusuf S., Eikelboom J., Oldgren J., Parekh A., Pogue J., Reilly P.A., Themeles E., Varrone J. (2009). Dabigatran versus warfarin in patients with atrial fibrillation. N. Engl. J. Med..

[25] Granger C.B., Alexander J.H., McMurray J.J., Lopes R.D., Hylek E.M., Hanna M., Al-Khalidi H.R., Ansell J., Atar D., Avezum A. (2011). Apixaban versus warfarin in patients with atrial fibrillation. N. Engl. J. Med..

[26] National Institute of Neurological Disorders and Stroke rt-PA Stroke Study Group (1995). Tissue plasminogen activator for acute ischemic stroke. N. Engl. J. Med..

[27] Hacke W., Kaste M., Fieschi C., von Kummer R., Davalos A., Meier D., Larrue V., Bluhmki E., Davis S., Donnan G. (1998). Randomised double-blind placebo-controlled trial of thrombolytic therapy with intravenous alteplase in acute ischaemic stroke (ECASS II). Lancet.

[28] Paciaroni M., Agnelli G., Falocci N., Tsivgoulis G., Vadikolias K., Liantinioti C., Chondrogianni M., Bovi P., Carletti M., Cappellari M. (2017). Early Recurrence and Major Bleeding in Patients With Acute Ischemic Stroke and Atrial Fibrillation Treated With Non-Vitamin-K Oral Anticoagulants (RAF-NOACs) Study. J. Am. Heart Assoc..

